# Editorial Correspondence

**Published:** 1878-06

**Authors:** Jas. H. Low

**Affiliations:** LaFayette, Texas


LaFayette, Texas, April 23d, 1878.

Dr. J. G. Westmoleland,

*My Good Friend*—Doubtless you will think that the old adage is verified, (especially in my case) "out of sight, out of mind," but I will dispel such thought from your mind by complying with a promise made to you before leaving Atlanta, that I would probably write you a short letter before my return to the Gate City.

After leaving Atlanta nothing occurred of any particular interest until our arrival at New Orleans, the great city of the Southwest; it appears to have a go-ahead-ativeness about it not surpassed by any city I have seen on my route, and in fact, equalled only by Atlanta. We visited many places of note about the city, and was not a little surprised to see the vast amount of business transacted and the improvements going on. The down-trodden city seems to be getting all right. We visited the French market, which excels the market of any city I have ever visited. You can hear nearly all languages spoken, see all colors of people, purchase anything in the fancy line from a spool of thread to a velvet robe, see all kinds of vegetables, all kinds of meats and get anything in the fish line from a craw fish to an alligator.

In close proximity to the market is Jackson Square and the old Spanish Cathedral; the former is visited daily by hundreds of the many strangers in the city, and is noted for its beautifully laid off walks and its artistically arranged flowers, and by an eqestrian statue, in the centre, of General Jackson, who looks probably as grand and daring as he did on the day he defended the Crescent City. The Cathedral is noted for its antiquity, being probably the oldest building in the city, and also for its beautiful paintings and frescoing. It is attended largely by the French.

I saw immediately one after the other three funeral processions, which led me to inquire of the gentleman who sat next to me in the street-car, (who, by the way, I supposed to be a medical man) in reference to the health of the city, who replied that the health of the city was excellent.

I visited several cemeteries, some of which were indeed beautiful—filled with elegant and costly monuments. We saw a very beautiful and elaborately finished monument in Greenwood Cemetery, erected to our Confederate dead, surmounted upon a square base, placed upon the four corners of which were the marble busts of Generals Lee, Jackson, Johnson and Polk.

At this season New Orleans is perhaps as healthy as any interior town. The time for malaria has not arrived, and as the epidemics to which she is subject depends upon this poison, I am not surprised to find the city comparatively free from disease. Yellow fever, that great bane of our Atlantic and Gulf coasts, is certainly the product of this insinuating morbific agent, much as has been said of importation. If it be the subject of importation, why should the sanitary means adopted last year prevent the ravages of this dreadful malady? Certainly the means were directed against the existence and dissemination of malaria in the city.

Our expectation of pleasure up the Red River would have been realized, had it not been for an occurence that took place aboard our boat shortly after leaving the city, a sadder scene than which I do not think I ever witnessed. Very early in the morning, after we left New Orleans, I heard the cry "My child—my child is dying!" and at that moment, before I and my wife had time to dress, there came rushing into our state room B—— N——, with his babe in his arms, in apparently a dying condition, but by the prompt application of such remedies as I happened to have, the child at last revived to some extent, when I, with another medical gentleman on board, used all our efforts to prevent a recurrence of the next paroxysm, but our efforts were unavailing, and in a very short time the child was attacked with another convulsion, and so on, one succeeding another in rapid succession, until at last death relieved the little sufferer just as we reached Baton

Rouge. We used all the remedies we had at hand, to-wit, baths, stimulating, friction, chloroform, etc.

Mrs. N. has lost two, and perhaps three, children the same way. Her children seem to inherit a disposition to convulsions of a fatal character.

The scenery up the Mississippi and Red rivers, in places, is beautiful and picturesque, dotted here and there with the old and dilapidated mansions of the once wealthy inhabitants. There is a sombre and gloomy appearance given to the lands on both banks of the river, by the large quantities of moss hanging from the branches and limbs of the timber. The doctors say the miasm rises as high as the moss grows, and no higher; and as the moss in some localities is much higher than it is in others, they contend the miasm will arise higher, spread farther, and be productive of much more sickness.

I again took railroad at Shreveport, and reached Texas on the ninth day after leaving Atlanta. Since my arrival I have been solicited to visit several cases of sickness, mostly fevers of the remittent and intermittent type—one case near me of a well-marked typhoid type, a lady of thirty-five years of age. There was much more torpor of the liver than usually attends that type of fever in Georgia, especially in the neighborhood of Atlanta. She had been laboring under the fever one week when I first saw her. Pulse about one hundred to the minute, tongue coated over, with edges and tip pale, (which, by the way, is the case with most of the tongues I have seen in Texas in all types of fevers) restless at night, to so great an extent, frequently, as not to sleep more than two hours during the night; loss of appetite. I found her in this condition when first called to her. I began my treatment with three grain doses of sub. mur. hydgr., repeated night and morning for two or three days, and then only one dose at bed time. In a few days her tongue commenced cleaning off. I then recommended calomel every other night, with quinine and whisky during the day—say two grains of the former three times daily. She continued to improve, rather slowly, I thought, though I concluded as they were the only remedial agents I had, and as she seemed to improve, I would let well enough alone. I had the satisfaction this morning when I visited her, to see her sitting up in a chair; and though she has been desponding most of the time, she acknowledges this morning that she will get well. She was clear of fever, tongue nearly cleaned off, and appetite fair.

I have been called in consultation to other cases, and have seen many of which I can give you a description when I return to the Gate City.

The mercury stands 90° Fahrenheit. Texas is a hot part of the world.

Respectfully your old friend,

JAS. H. LOW, M.D.

---

